# Zoning Optimization Method of a Riverfront Greenspace Service Function Oriented to the Cooling Effect: A Case Study in Shanghai

**DOI:** 10.3390/ijerph192316191

**Published:** 2022-12-03

**Authors:** Yunfang Jiang, Xiaolin Li, Jing Huang

**Affiliations:** 1 School of Urban and Regional Science, East China Normal University, Shanghai 200241, China; 2 The Center for Modern Chinese City Studies, East China Normal University, Shanghai 200241, China; 3 Future City Lab, East China Normal University, Shanghai 200241, China; 4 Shenzhen Overseas Chinese Town Middle School, Shenzhen 518053, China

**Keywords:** urban heat island (UHI), blue–green space, riverfront area, spatial morphology, urban cooling effect (UCI), boosted regression trees (BRT), marginal effect (ME), Shanghai

## Abstract

Blue-green space commonly provides multiple ecological service functions, especially thermal environment comfort for citizens. The greenspace of the riparian buffers along 22 river channels in Shanghai was selected as the study object, and remote sensing and GIS technologies were used to obtain the quantitative composition and morphological indices of riverfront greenspace and the spatial distribution data of the land surface temperature in the study area. Through BRT modelling and statistical analyses, the interactive correlations among the three aspects, namely, the spatial patterns of riverfront greenspace, their specific functional zoning, and cooling island differentiation characteristics, were explored. The results showed that different river types served for different functional zones of the city, namely, high-density built-up zoning, new urban-growth zoning in built-up areas, suburban areas, and rural areas, and had specific regular patterns of morphosis and service function of riverfront greenspace. These also led to a significant spatial differentiation pattern of cooling intensity levels, which generally appeared in the approximate circle differentiation structure of the cooling island in the city riverfront area. The study further proposed the key factors and corresponding strategies for optimizing the greenspace pattern to strengthen the cooling intensity levels of different river types. This study summarizes the landscape composition paradigm of riverfront greenspaces at the urban mesoscale and provides adaptive planning methods for better local microclimate conditions.

## 1. Introduction

Waterfront greenspace is the interlace and buffer zone between land space and water space. It has both natural ecological elements and artificial landscapes. It is an important corridor for energy flow and species circulation in the ecosystem and an important place for residents’ leisure activities, which often constitutes the most vital open space in the city [1,2,3]. Urban riverfront greenspace meets the leisure needs of residents with its superior hydrophilicity and comfort and provides important natural ecological services [4]. Research on the optimization of the ecological pattern function of urban waterfronts [4,5,6,7,8,9] and the improvement of microclimates by blue-green space has become a focused area of many disciplines.

Blue-green space has a SCE due to a combination of waterbodies and vegetation. Riparian high-density vegetation affects the solar radiation balance and turbulent energy of the water body to adjust the water temperature, promote air convection, and reduce the water temperature of the water bank [10]. Additionally, the evapotranspiration of water bodies is stronger under the influence of greenspaces [11]. The shading and transpiration of the canopy of riverfront trees are conducive to cooling and humidifying [12]. The greenspace adjacent to the river can generate a greater CE (CI) [13]. A certain width of green belt on both sides of the river helps to improve the local microclimate and to effectively reduce the environmental temperature by 5–10 °C [10]. The CE of greenspace in the waterfront area is obviously greater than that of a single waterbody or greenspace [14].

The CI of blue–green space in the riverfront area is affected by greenspace morphology. Greenspace morphological factors often include the area scale, vegetation coverage, and landscape shape index. Greenspace with a higher percentage of green coverage would provide stronger shading and transpiration processes of greenspace and a stronger CI. Green coverage, which is generally evaluated by the normalized difference vegetation index (*NDVI*) or fractional cover values of vegetation space (Fv), is negatively correlated with the land surface temperature (LST) in summer [15,16,17,18,19]. The CI of riverbank belts with high vegetation coverage is much stronger than that of flinty riparian. High vegetation coverage can strengthen the cooling of riverbanks, and the maximum cooling occurs in places with a large number of trees covering and shading both sides of the banks [20]. The greenspace network mode with high connectivity is conducive to the cold air transportation of urban rivers to produce a better CI [21,22]. Interconnected greenspaces provide a higher CI to adjacent areas [23,24]. Increasing the cohesion degree of greenspaces is also conducive to enhancing the CE [23], and the LST values are negatively correlated with the aggregation index, which is another connection degree index [24]. LST values are positively correlated with the LSI index of green patches, and green patches with simple shapes and high concentrations have lower LST values [24]. In addition, the object material with high surface albedo will lead to a lower level of solar energy absorption, thus reducing the surface and adjacent air temperature [25]. The surface albedo is to a certain extent negatively correlated with the LST [26].

The SCE in the riverfront area is also affected by the spatial factors between blue space and greenspace. The blue-green spatial factors mainly include river morphology, distance to the riverbank (D), and the connectivity of blue-green space [18]. The water surface ratio (Wr) of a riparian area affects land surface temperature (LST). There is a negative correlation between Wr and LST [27], namely, the surface temperature gradually decreases with increasing Wr [28]. Combined with the findings from relevant studies of built-up areas (BUA) in South China, the Wr will also affect the space humidity, thus affecting the thermal environment of the space. When Wr is 5%, the relative humidity in the downwind direction area is 5~8% higher than that in the upwind direction area [29]. D is another important factor affecting the distribution of urban heat islands (UHIs) [21]. For every 1000 m increase in distance from the river, the UHI decreased by 0.6 °C in the summer [30]. A river with a width of 35 m can lead to a decrease of approximately 1–1.5 °C in the ambient temperature, and the decrease can be increased if greenspace is present on both sides of the river [31]. The larger the river width is, the stronger the ability to alleviate the thermal environment [32]. The dominant wind direction is also an important factor providing more cooling for the area in the downwind direction [33]. The prevailing wind direction can promote air circulation and further enhance the CI [34].

In studies on the influencing factors of blue–green spaces on the CI, field measurement, remote sensing technology, and numerical simulation methods are usually used. The field measurement method studies the CI of blue–green spaces by obtaining the temperature data of local specific points [20,35]. The microclimate is monitored with sensors and combined with the temperature and other data extracted from the meteorological station. Using this method, it was found that the temperature of the land surface corresponds exactly to the distinct land cover types. The temperature of water bodies and vegetated area ranges between 12 and 14 °C and between 8 and 10 °C lower than the LST of roads and built-up areas in summer and winter times, respectively [36]. However, the measured workload is large and there are many interference factors that affect the measurement results. Numerical simulation is a three-dimensional dynamic simulation based on computational fluid mechanics. At present, the commonly used software platforms are ENVI-met, fluent, WRF, Airpark, etc. Among them, ENVI-met is widely used to simulate the microclimate of different pattern of blue–green spaces [21,22]. However, the maximum simulation grid scale of ENVI-met software is limited to 2 km × 2 km, which is more suitable for the microscale [18,37]. The technology of RS and GIS can provide continuous LST data at the macroscale and can also provide overall data for urban mesoscale research [37,38,39]. Based on LST data obtained from ASTER image, the impact of surrounding land types on the cooling of urban-park greenspace was studied, and the research found that the business district would block the extension of the park for cooling, and the valley terrain would help to transport the park’s cold air to the surrounding areas [40]. However, the landscape composition of ecological land has more influence on LST values than the spatial allocation [39]. Therefore, remote sensing technology can solve the problem of spatiotemporal discontinuity of on-site monitoring data by efficiently obtaining a large range of LST data in the study area.

The focus of research on the cooling island effect is gradually shifting toward more complex built-up space environments. In terms of the river morphological factors affecting the CI, studies have shown that the width of the river and the land use along the riverside are the two fundamental factors [32,41]. Additionally, studies have demonstrated that the width classifications of urban-rivers buffer areas should be identified by the riverfront development regulation and land use in waterfront areas [18,42]. Moreover, the ecological service function of urban river corridors is related to the width of the corridor and its internal structure. The width of the riverbank and the watershed corridor should be approximately 402–1609 m [43]. When assessing the visual diversity (natural richness) and visual disconnection (anthropogenic impact) of the landscape, the grade of the homogeneous landscape should be 1000 m and the comparatively diverse landscape should range between 1000 and 3000 m [44]. Thus, from the perspective of a landscape thermal regulation function and ecological integration structure, the width of an urban-river-related functional region should be approximately 1000–3000 m in an optimal state.

Remote sensing technology has laid a technical foundation for the quantitative analysis of thermal environmental effects of urban land-use types. In the study process of the emerging spatial-pattern factors affecting the urban climate environment, as a rational development supporting decision-making, it is obviously more important to innovate the research methods of spatial data for facing physical objects. Based on remote sensing technology and GIS spatial-analysis methods, discovering the composition regulation in the sense of greenspace typology and introducing the correlation characteristics between the quantitative description factor characteristics of greenspace types and the intensity of greenspace CE into the larger-scale level on the riverfront spatial composition regulation, so as to solve the specific object optimization issues that planning needs to face, has obviously become more urgent. Compared to previous spatial-analysis methods that use segment pixels as the research object, this study uses greenspace types as the research object. This transformation of the space unit can provide a rational development planning strategy to directly explore and optimize the greenspace pattern.

Exploring the regional service characteristics of riverfront greenspace and studying how its spatial structure affects the CI distribution will play a guiding role in greenspace system planning based on spatial function optimization. In combination with the current situation of waterfront greenspace construction in Shanghai, this paper studies the dominant characteristics of greenspace patterns in the riverfront area from the perspective of different spatial functional partitions of urban development. Through correlation analyses between quantitative indices of the greenspace spatial pattern in the riverfront area and the LST distribution, the cooling mechanism and the essential factors for riverfront greenspace optimization were determined, and differential development strategies of the greenspace pattern in various types of rivers are proposed. This study provides an important analysis method and rational development basis for improving the comfort of the urban thermal environment and forming a better SCE of blue-green space.

## 2. Study Area and Methodology

### 2.1. Study Area

**Figure 1:**
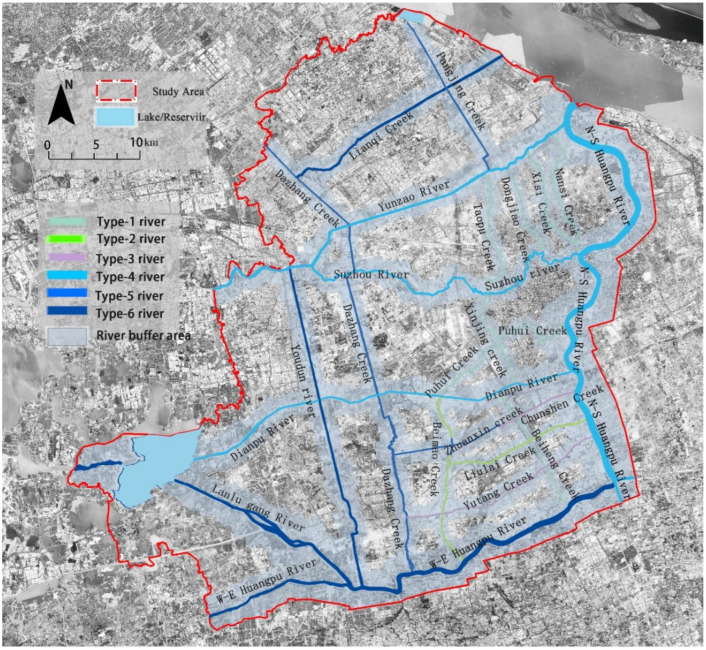
Raster image of the study area and location of the backbone river corridors in Shanghai, China.

Shanghai is located between 30°40′ and 31°53′ N latitude and between 120°52′ and 122°12′ E longitude in the Yangtze River Delta, with a subtropical monsoon climate. There are many rivers and lakes in the city, with rich water resources and a dense river network. A relatively holistic area, enclosed by the west–east and north–south segments of the Huangpu River in Shanghai, was selected as the study area. In the study area, the water system of the backbone rivers was considered based on the “2017 Shanghai River Channel (Lake) Report” [45], and the river buffer area was identified as the riverfront study area (Figure 1). The study was conducted in a high-density area, with a high-land-use intensity and a very obvious difference in the spatial distribution of the thermal environment. Additionally, the urban river system in the region is relatively complete, and the river width is quite different. Various river corridors provide many greenspaces, and the greenspace types are diverse, which provided a good case area for studying the CI effect in urban riverfront areas.

Different buffer zones were delimited according to river width grade. It has been found that the CI threshold distances were 550 m, 780 m, 1000 m, 1500 m, and 1700 m in different river regions of Wuhan city [44]. Furthermore, the threshold distances of Shanghai have revealed that, when the river widths were 30 m, 30–50 m, 50–70 m, and more than 70 m by the technical standard for the river classification of plain river networks [46], correspondingly, the maximum impact distances were about 600 m, 900 m, 1200 m, and 1395 m, respectively [18]. The urban backbone rivers in the study area were divided into small/medium-sized backbone rivers with widths of less than 30 m and regional backbone rivers with widths of more than 30 m. Subsequently, corresponding to the implementation and management orientation, the river buffer boundaries were combined with the size of the complete urban block and the road boundary line. Finally, buffer zones with widths of 500–800 m and 800–1500 m were identified according to two grades of river width. Additionally, in consideration of the need to meet the overall development management of the urban riverfront area, the buffer zone of the Huangpu River system was bounded by the regional planning scope by the “Huangpu River waterfront area construction plan (2018–2035)” [47].

The space utilization of the river corridor areas was closely related to the functionality of surrounding land development. Therefore, the spatial heterogeneity of the riverfront area presented zoning characteristics by serving different city functions. Zonings specifically included the following: high-density core area, other BUAs (newly developed areas in the south section of the built-up area), suburb (urban-rural fringe), and rural. Based on different development zoning and river-network conditions in the study area, this study further defined six river types in the study area to further analyze the dominant characteristics of their riverfront greenspace composition and the intensity distribution of the CI effect. The distribution of these six river types is shown in Figure 1.

### 2.2. Research Framework

**Figure 2:**
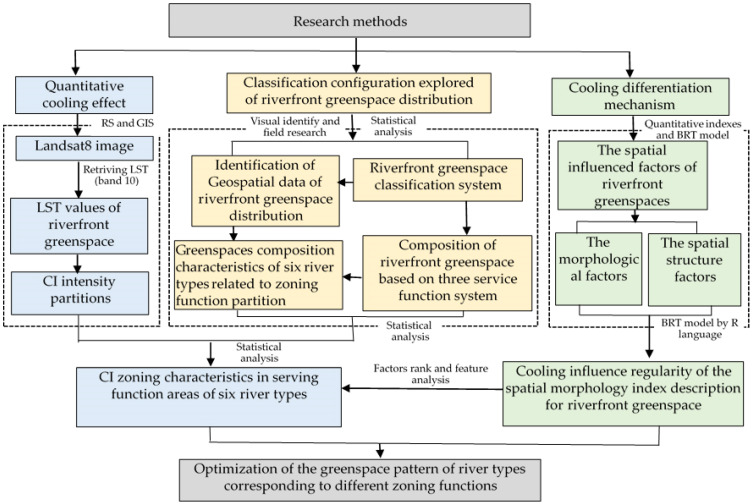
Framework flowchart of the study.

The methodological framework of this study is shown in Figure 2. The typical process for CI research involves four key steps: (1) Quantitative CE. Extraction of the urban surface temperatures based on band 10 of Landsat 8 remote sensing images and the CI intensity distribution of greenspaces with temperature difference intervals were obtained. (2) Classification configuration exploring riverfront greenspace distribution and establishment of a geospatial database of the river corridors and riverfront greenspaces. High-resolution aerial image data and administrative topographic vector data were combined with manual field surveys to establish a spatial distribution database of greenspace types based on three service function systems; composition of riverfront greenspace at regional level and the river reach level were statistical analyzed. The dominant distribution features of riverfront greenspace types were conducive to the forming of six river type classifications associated with zoning partition. (3) Studying the cooling differentiation mechanism. The spatial influenced factors of riverfront greenspaces were quantified, and the greenspace morphological index and spatial structure factors system were used to describe each patch feature of riverfront greenspace. Correlation analyses between the LST values and quantitative indices of the blue–green space were used to reveal the cooling influence regularity of the spatially influenced factors of the different riverfront green patches. (4) Finally, discovering the regularity between the riverfront greenspace types of six river types and the CI distribution characteristics, and proposing the optimization strategy of the greenspace pattern of river types corresponding to different zoning functions. CI zoning characteristics in serving function areas of river types were produced by the composition and spatial morphology of each type of riverfront greenspace. Dominant characteristics of the riverfront-greenspace-types composition related to the zoning function of river types are determined using factor-rank and feature analyses.

### 2.3. Land Surface Temperature (LST) and Cooling Island (CI) Intensity

LST inversion was performed on envi5.3, a remote sensing image processing software. The remote sensing image data used was the Landsat 8 satellite image that was taken by the United States Geological Survey (USGS) at 10:25 on 24 August 2017, and the cloud coverage was 0.4%. This process was conducted in two steps. First, the band 10 thermal infrared data of Landsat 8 thermal infrared sensor (TIRS) images were subjected to radiometric calibration and atmospheric correction based on the fast line-of-sight atmospheric analysis of spectral hypercubes (FLAASH) model in the ENVI software. Second, band 10 of the TIRS image was used to retrieve the land surface temperature (LST). Because the parameters, such as atmospheric transmittance and atmospheric average temperature of the single-window algorithm, were difficult to obtain, the water vapor content in Shanghai was large, and the single-channel algorithm was greatly affected by the atmospheric water vapor content [48], which is not applicable to Shanghai. This study adopted the method of radiative transfer equation inversion to obtain the LST value; the accuracy of this inversion algorithm is higher than that of other algorithms [49]. The calculation process was as follows:

First, the variable’s land surface emissivity, namely, *ε*, can be calculated as follows:

(1) ε=0.004Fv+0.986

(2) $$Fv=\left[\left(NDVI-NDVI_{soil}\right)/\left(NDVI_{veg}-NDVI_{soil}\right)\right]$$

where *Fv* is the fractional cover value of the vegetation space; *NDVI* is the normalized difference vegetation index; *NDVI_Soil_* is the *NDVI* value for areas that have completely bare soil or no vegetation cover. According to the calculated *NDVI* data, we used the cumulative percentage of *NDVI* in the compute statistics tool of ENVI5.3 software, so as to take *NDVI* with 95% confidence as *NDVI_Veg_*, *NDVI_Veg_* = 0.666697, and *NDVI* with 5% confidence as *NDVI_Soil_*, *NDVI_Soil_* = −0.184233. The influence of the atmosphere on the surface thermal radiation was estimated.

Second, the atmospheric influence was subtracted from the total amount of thermal radiation observed by the satellite sensors to obtain the surface thermal radiation intensity. The radiation brightness of the black body was calculated. The calculation formula is as follows [50]:

(3) Lλ=[εB(TS)+(1−ε)L↓]τ+L↑

where *Lλ* is the luminance value of the thermal infrared radiation received by the satellite sensor, *L*↑ is the atmospheric upwelling radiance, *L*↓ is the atmospheric downwelling radiance, *ε* is the land surface emissivity, *TS* is the true land surface temperature, *B*(TS) is the black-body radiation intensity determined using the Planck radiation function, and *τ* is the atmospheric transmissivity. The three atmospheric profile data points (*τ*, *L*↑, and *L*↓) can be obtained from NASA’s website (http://atmcorr.gsfc.nasa.gov, accessed on 25 January 2020).

In the formula, *B*(*TS*) and *TS* can be calculated as follow:

(4) B(TS)=[Lλ−L↑−τ(1−ε)L↓]/τε 

Third, the surface temperature was retrieved. After obtaining the radiance of the black body with temperature *TS* in the thermal infrared band, the real surface temperature *TS* was obtained according to the inverse function of Planck’s formula. The formula is as follow:

(5) TS=K2/ln(K1/B(TS)+1)

where *K*1 and *K*2 are constants. For the TIRS data of Landsat-8, *K*1 = 774.89 (mWm^−2^sr^−1^μm^−1^) and *K*2 = 1321.08 (mWm^−2^sr^−1^μm^−1^).

**Figure 3:**
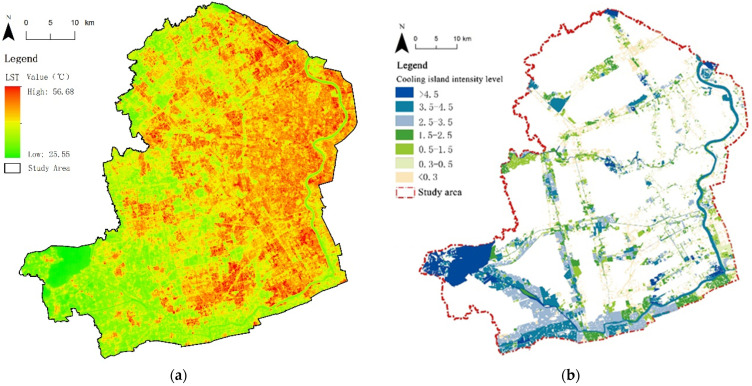
Distribution of LST values and CI intensity in the study area. (**a**) LST distribution; (**b**) CI intensity level distribution.

The retrieved surface temperature of the main urban area of Shanghai (Figure 3a) was input into the ArcGIS platform, and the average surface temperature data of each green patch were counted using the zonal analysis tool of ArcGIS.

CI intensity was selected as the quantitative standard of the CE in this study. The CI intensity of greenspace was calculated using the temperature difference between the average LST of each green patch and the average LST of the area where the green patch was located. The CI intensity was divided into seven levels with temperature difference intervals of less than 0.3 °C, 0.3–0.5 °C, 0.5–1.5 °C, 1.5–2.5 °C, 2.5–3.5 °C, 3.5–4.5 °C, and greater than 4.5 °C to obtain the CI intensity distribution of greenspaces (Figure 3b) in order to analyze and compare the CE of greenspaces under different urbanization and development construction backgrounds.

### 2.4. Classification System of Riverfront Greenspace

The spatial composition and structure of urban riverfront greenspace constitute the urban ecological pattern of the city. A green corridor system along urban rivers provides the natural physical space to support ecological security. As a subsystem of the urban greenspace system, the study of the internal space feature of riverfront greenspaces should be assessed by a detailed classification system of greenspaces. To provide operability of spatial development and implementation management for riverfront land use, the concept category of generalized greenspace was adopted. The final classification system adopts the configuration method of “functional system class + subdivision greenspace type” [51,52].

First, the functional system was divided into three greenspace classes according to their service functions: (1) ecological conservation land, the main service function of which is to provide water sensitivity reservation and protection in the natural state of the riverfront; (2) recreational greenspace, the main function of which is to provide social recreation and leisure services for residents’ lives; and (3) agricultural use area, which is to provide productive plantations needed for residents’ production and living.

**Figure 4:**
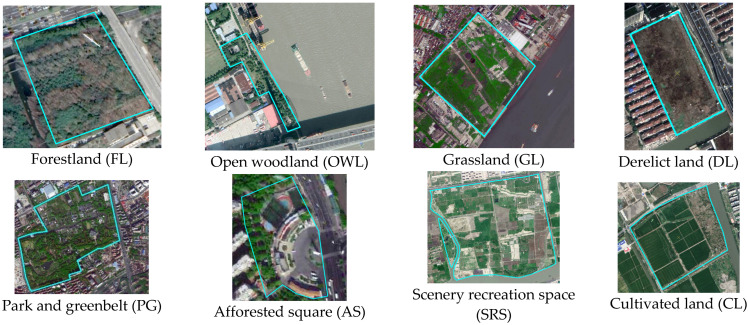
Identification of greenspace types and morphological characteristics in the riverfront area.

Second, broad types of greenspace according to two national standards, namely, the “Land use status classification (GB/T 21010–2017)” [51] and the “standard for classification of greenspace (CJJ/t85-2017)” [52], were subdivided into 8 types: forestland (FL), open woodland (OWL), grassland (GL), derelict land (DL), park and greenbelt (PG), afforested square (AS), scenery recreation space (SRS), and cultivated land. The classification framework and morphological characteristics of each type are shown in Table 1 and Figure 4.

### 2.5. Spatial Data and Morphology Quantification of Riverfront Greenspace

**Figure 5:**
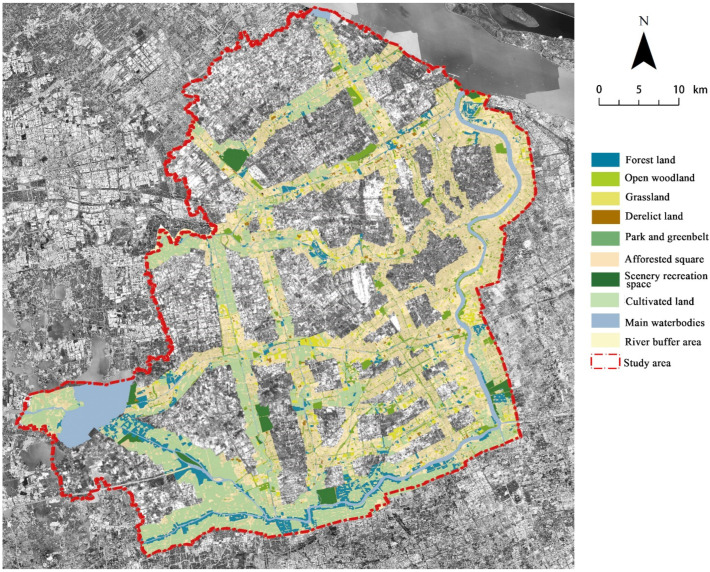
Distribution of greenspace types in the study area based on land-use classification.

Using the high-resolution image downloaded from Google Earth in 2017 and referring to the topographic vector data of the Shanghai road network with a scale of 1:50,000 obtained through investigation from the municipal bureau of planning and natural resources in 2015, the ground control point GCP was selected for geometric fine correction on the ArcGIS software platform. The image recognition of the river network and greenspace adopted the method of combining aerial photograph interpretation with supplementary field investigation. Finally, the spatial distribution of the main water-body network and 4550 patches of greenspace in the study area were obtained (Figure 4 and Figure 5).

The characteristics of greenspace landscape patterns in waterfront areas can be described quantitatively from the area scale of green patches, vegetation coverage, related landscape pattern indices, and the spatial structure relationship between green patches and rivers. In combination with the previous study results on relevant CI-influencing factors of blue-green space, this study selected 8 indicators to describe the spatial characteristics of the blue-green space system in the riverfront area (Table 2).

(1) Spatial Morphological Variables

a. Area

The area of the green patches was calculated using the ArcGIS10.4 software platform.

b. Fraction of the vegetation coverage (Fv)

Fv describes the greenness of a green space. The value represents the percentage of the vertical projection area of the vegetation (including leaves, stems, and branches) on the ground to the total area of the statistical area and is a key parameter to describe the vegetation coverage on the ground [53,54]. Fv is the percentage of vegetation reflection in a pixel to the total reflection by decomposition from the interior of a single pixel [55]. The calculation formula is presented as formula (2).

c. Landscape shape index (LSI)

The landscape shape index (LSI) represents the boundary shape of the greenspace and is determined by calculating the deviation between the shape of a greenspace patch and a square of the same area [56]. The calculation formula is as follows:

(6) $$LSI=\frac{0.25L}{\sqrt{A}}$$

where *L* is the total perimeter of the green patch and *A* is the area of the green patch.

d. Albedo

The surface albedo is the ratio of the surface reflection flux to the incident solar radiation flux on the surface of a greenspace [57,58]. Albedo reflects the comprehensive heat radiation impact of vegetation coverage in greenspace and the three-dimensional shape of adjacent surrounding environment on greenspace [59]. In this study, the inversion model for Landsat-TM data established by Liang was applied to retrieve the Landsat 8 data to estimate the surface albedo [60]. The calculation formula is as follows:

(7) $$Albedo=0.356B_{2}+0.130B_{4}+0.373B_{5}+0.085B_{6}+0.072B_{7}-0.0018$$

where *B*2, *B*4, *B*5, *B*6, and *B*7 represent the blue, red, near infrared, and 1 and 2 bands of Landsat 8 data, respectively.

(2) Spatial Structural Variables

e. Cohesion

The degree of patch aggregation is an effective index to evaluate the connectivity of landscape spatial structure characteristics. The cohesion index was selected to express the connectivity of each green patch in the holistic blue-green ecological network, so as to measure the connectivity of the natural state of greenspace. As the distribution of landscape patches becomes more concentrated and the natural connectivity increases, the cohesion index becomes higher [61]. C values were calculated using fragstats4.2 software, and the calculation formula is as follow:

(8) $$Cohesion=\left[1-\frac{\sum_{i=1}^{m}\sum_{j=1}^{n}P_{ij}}{\sum_{i=1}^{m}\sum_{j=1}^{n}P_{ij}\sqrt{a_{ij}}}\right]{\left[1-\frac{1}{\sqrt{A}}\right]}^{-1}\times 100$$

f. Location of the greenspace (LG)

This variable refers to the distribution position of greenspace in a city zone relative to rivers [14,18]. It was defined according to the location of greenspace in given urbanization zoning and the dominant wind direction in summer to determine the position of greenspace relative to the river. Specifically, it can be divided into four types of greenspace distributed in the windward direction of the built-up area, the leeward direction of the built-up area, and the windward and leeward directions of the rural area.

g. Water surface ratio (Wr)

The Wr refers to the ratio of the area carrying water functions within a certain area to the area’s total area [62]. The values were calculated by the percentage of water surface area in each partition area in Arc ArcGIS10.4 software. The Wr index can be further subdivided as follows according to waterbody composition:

Water surface ratio of main backbone rivers (Wr-m): percentage of the main rivers’ surface area adjacent to a greenspace in the area of the corresponding riverfront blocks.

Small water surface ratio (Wr-s): percentage of small waterbodies and branch rivers around the greenspace in the area of the corresponding riverfront blocks.

h. Distance to the riverbank (D)

This value is the smallest geometric distance from the green center to the riverbank. The geometric center point of the green patch was extracted using ArcGIS10.4 [63]. Subsequently, the near analysis tool in ArcGIS10.4 was used to obtain the distance from the river.

### 2.6. BRT Model Analysis and Statictic Analysis

There is an influence correlation between the morphology variables of greenspace and the LST values. However, this correlation is nonlinear. The boosted regression trees model (BRT model) is a research method of machine learning that combines the advantages of the regression model and growth model [64] and can deal with nonlinear variables and interaction effects between variables. The model is obviously different from the traditional linear regression model. The BRT model has been widely used in urban studies, such as those investigating the reactive relationship between urban expansion and its influencing factors [65], and in identifying the most important predictor of cold air paths [66]. In recent years, the BRT model has also been applied to the study of the relationship between the urban heat island effect and urban two-dimensional and three-dimensional indices [67].

The BRT model was used to explore the correlations and interactions between various spatial factors of green patches and the thermal environment. In the model, the causal variable was the LST values of green patches and the independent variable was the descriptive spatial variables. The parameters of the model were set as follows: the complexity of the decision tree was 5, the learning rate was 0.01, the bag fraction was 0.5, and the data type was Gaussian, based on previous research [66,67]. The model randomly extracted 50% of the data for analysis, with 50% of the data used for training each iteration, and the 10-fold cross-validation method was used to compare the prediction performance of the model.

In terms of the output results of BRT model, first, it is possible to estimate the relative impact (or contribution) of each variable in proportion and add the sum to 100. Second, it can also obtain the marginal effect (ME) curve. Based on the ME curve, the threshold value of the correlation effect of the independent variable on the causal variable was analyzed. The ascending trend of the curve indicates that the factor was positively correlated with the causal variable; otherwise, it was negatively correlated. The degree of inclination of the curve indicates the strength of the influence.

According to the grade interval difference of greenspace morphology data in the study area, combined with the spatial factor threshold of the green patches for cooling efficiency maximum obtained by BRT model analysis, this study identified the grade interval and factor rank of conventional spatial quantitative indices, and finally described the greenspace type features through the grade rank classification in the study area to discover the regularity of the combined effect of various factors. The main classification of spatial indices of greenspace can be classified as shown in Table 3.

Using statistical methods, the proportion of the total space composition of the riverfront greenspace in the study area was analyzed, and the spatial characteristics of the riverfront greenspace pattern in the study area were obtained; the proportion of greenspace composition of buffer areas in six river types within different service subareas was counted to summarize the dominant characteristics by its composition; through classified statistics assessing relationships between different CI intensity grades and greenspace type composition, the composition of dominant greenspace types corresponding to the CI intensity at all levels were found, and the main distribution regularity of various types of greenspace in six river types generated different CI grades, so as to find the existing development problem of improving the CE of greenspace systems in different service functional zonings.

## 3. Results

### 3.1. CI Distribution Characteristics of Greenspace Patterns

#### 3.1.1. LST Difference of Various Greenspace Types

**Figure 6:**
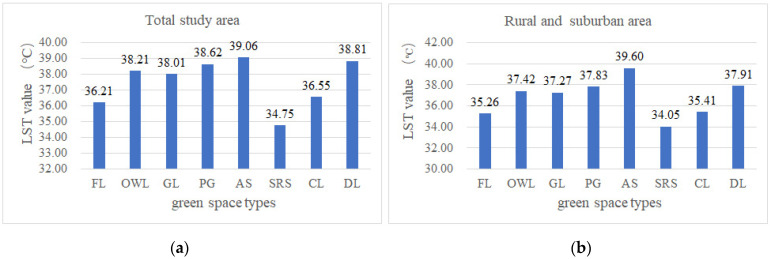
The mean surface temperature of different greenspace types. (**a**) The values in the study area; (**b**) the values in rural areas and SUA.

According to the spatial classification statistics of various types of greenspaces, the mean surface temperature of different greenspaces was obtained (Figure 6a,b). SRS and FL types were the types of greenspace with low mean LST due to their generally high vegetation coverage; CL type in the study area had the characteristics of being concentrated densely on large-scale distribution, and mainly located in the suburbs with a low mean LST; PG type specifically included parks of different grades, belt-shaped public greenspaces along roads or water systems, and neighborhood parks in some blocks. There were many low-value vegetation coverages in the PG type, and the LST changes of this type were obvious, causing a higher mean LST of this type. The OWL type also presented a high average surface temperature, as it was basically located in the built-up area, and the patch areas were mostly small. AS and DL had very high LST values.

#### 3.1.2. The Area Proportions of CI Intensity of Different Types of Greenspace

From analyzing the area proportions of each greenspace type in each level of CI partition (Table 4), a large area proportion of the FL type was found, which belonged to level-six, level-five, level-four, and level-three CIs. The CI distribution of the three types, e.g., OWL, GL, and PG, was relatively consistent. Most of them belonged to insignificant CIs, and larger area proportions were level-two and level-three CIs. The AS type could achieve a level-four CI when the riparian vegetation coverage was seldom high, but most of the AS type was at an insignificant CI level. A large proportion of the SRS type was at a high CI level, and the green patch with a low-level CI was mainly due to the low internal vegetation-coverage ratio and excessive hard-surface project. The CL type had CIs mostly at levels four and five. More than 2/3 of the DL type was comprised of an insignificant CI.

#### 3.1.3. Spatial Differential Characteristics of CI Distribution

**Figure 7:**
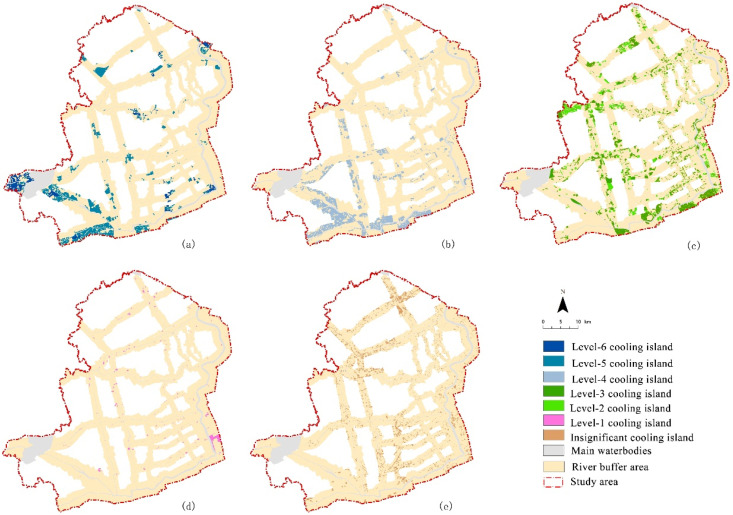
Spatial distribution of CI at different levels. (**a**) Level-six and level-five CI; (**b**) level-four cold island; (**c**) level-three and level-two CI; (**d**) level-one CI; (**e**) insignificant CI.

Through the hierarchical visualization of the CI intensity in the riverfront area, it was found that the CI pattern of blue-green space presented significant urban spatial differentiation characteristics (Figure 3b and Figure 7). The CI pattern was similar to circle differentiation structures, which was related to the river distribution with different zoning. From RA to the built-up area, the intensity grade of CI changed from high to low. A large number of CL patches, large-scale FL, and SRS were located in the suburbs, resulting in the high CI effect. The PG and other ecological-conservation functional patches in a small-scale scattered form were more concentrated in highly concentrated built-up areas. Their CIs were affected by their own morphology and the surrounding urban heat island, which led to low CI intensity partitions.

According to the statistics of the composition of greenspace types with different CI intensity levels, it was found that the main greenspace type composition corresponded to CI intensity at different levels (Table 5, Figure 7).

Level-six and level-five CIs were composed mostly of FL, SRS, and CL types. These green patches of CL, SRS, and FL types have a certain large-scale and concentrated distribution feature to provide strong riverfront CIs (Figure 7a). The green patches corresponding to level-four CIs were mostly CL, FL far from rivers, and PG with moderate area scale (Figure 7b). The level-three and level-two CIs were essentially composed of CL (isolated large-scale CL patches), and FL, PG, and large-scale GL also mostly belonged to the distribution area of these two CIs (Figure 7c). The level-one CI mostly corresponded to CL type under the influence of a specific built-up environment, small-scale PG, and SRS adjacent to the built-up area. Among the three types of greenspace, OWL, GL, and DL, a large proportion of these three types of green patches also belonged to level-one CI (Figure 7d). The composition types of greenspace with insignificant CI were relatively diverse and comprised most of the DL, PG, OWL, GL, AS, and other greenspace types. PG type in the high-density built-up area or in an industrial zone with a small-scale area, PG along main road, and remnant small-scale CL in the SUA all did not have significant CI effects (Figure 7e).

### 3.2. Composition of Riverfront Greenspace

#### 3.2.1. Holistic Composition Characteristic of Riverfront Greenspace

The proportions of various types of greenspace were counted, and the characteristics of greenspace composition in the riverfront area were analyzed (Table 6). Among all landscape types of greenspace in the riverfront area, the area proportions of four types, e.g., CL, FL, PL, and GL, were comparatively large.

CL accounted for the largest proportion of the total greenspace area, exceeding half of the total greenspace area. The proportions of FL and PL were similar. According to the analysis of greenspace class based on service function, the proportion of agricultural use area was 55.19%; the proportion of ecological conservation land was 26.76%; and recreational greenspace was 18.05%. The proportions of the three greenspace classes showed that the public activity function of the riverfront space landscape in the study area was relatively weak. However, the proportion of grassland and derelict land in various types of riverfront greenspace was approximately 9.36%, which demonstrated to a certain extent that there is great potential for the riverfront area to utilize and improve the service function of greenspace.

#### 3.2.2. Spatial Differentiation Characteristics Based on Service-Function Zoning

**Figure 8:**
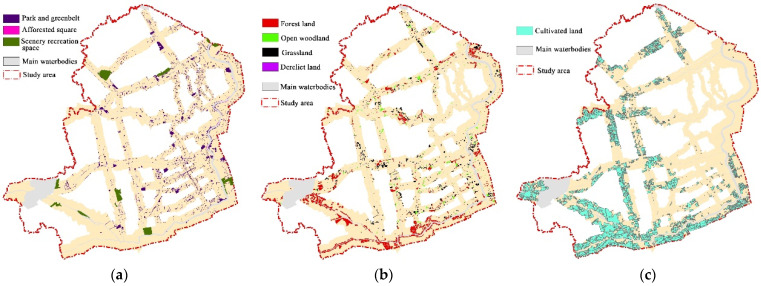
Distribution pattern of three classes of greenspace in the study area based on service function. (**a**) Recreational greenspace system; (**b**) ecological conservation land system; (**c**) agricultural use area system.

The distribution of recreational greenspace in the riverfront area was relatively scattered (Figure 8a), which was significantly related to the spatial pattern by which the river flows. In the built-up area, especially in the several rivers within the high-density built-up area, the PG type was densely distributed, and the waterfront green corridors were continuously connected, with a certain width scale. In the suburbs, the large-scale recreational greenspaces were the main type of greenspace, the distribution was relatively balanced to a certain service area, and all of them were connected with the river network.

Greenspaces with ecological conservation functions were distributed largely in the suburbs and rural areas wherein rivers flow (Figure 8b), and the average area of the green patches was relatively large. In particular, the west–east orientation segment of the Huangpu River, as an important water source location in Shanghai, formed a certain ecological protection pattern with forestland continuously along the riverside area. The forestland of other rivers was still distributed mostly in an isolated pattern in the suburban segment. In the high-density built-up zoning (HDZ), there were only a few small-scale green patches of these types. In the new urban growth zoning (NGZ) of the built-up area, the patterns of FL, OWL, and GL presented diversified agglomeration.

Greenspaces with agricultural-use areas were concentrated in SUA and RA (Figure 8c). There were still small-scale scattered CL patches in the riverfront of NGZ.

In general, the heterogeneity of greenspace patterns in the riverfront area has a certain regular zoning pattern. In the high-density built-up area, there was a relatively mature recreational greenspace system to serve the residents’ leisure requirements. In NGZ and SUA, there were diversified greenspace types, and ecological protection and land use development were both emphasized. The greenspace for agricultural use in the SUA was significantly higher than that in NGZ of the city. In the area with rural river segments, agricultural production and ecological protection were the two main functions. Due to the multicenter pattern of land use in the Shanghai metropolitan area, a certain number of rural large-scale recreational greenspaces were built within the reach region of new town construction.

#### 3.2.3. Greenspace Composition of Different River Types

By analyzing the spatial distribution of greenspace types in the riverfront area (Figure 5 and Figure 8), it was found that the distribution of riverfront greenspace types has the differentiation characteristics under the influence of development intensity in different locations. The dominant composition characteristics of greenspaces in different river-type zonings are show in Table 7.

Type-1 rivers were mainly located in the high-density built-up area, with a width of less than 30 m. The PG type of riverfront greenspace accounted for a large proportion, and public leisure and recreation were the dominant service functions. The area percentage of this type of greenspace exceeded 50% (Table 8), and the river in the central activity area had basically been built into an open greenbelt landscape.

Type-2 rivers were located in the NGZ of the built-up area. The river width was also less than 30 m. The composition of greenspace in the riverfront area presented multiple service functions, and the types of greenspace with a large area proportion were CL, FL, and GL and newly built PG types;

Type-3 rivers were located in the NGZ of the built-up area, with widths greater than 30 m and their riparian space was planned to be landscape corridors. The surrounding tributaries of this type are rich, and the Wr-s was high. Combined with the water surface, this type of river was planned to be constructed by the public leisure service function. Therefore, waterfront parks accounted for a relatively large proportion. There also existed a large proportion of GL and CL types of greenspace in the riverfront area.

Type-4 rivers were cross-district, crossing the built-up areas (BUAs), suburbs, and rural areas with widths greater than 30 m. The riverfront area was guided by the construction of multifunctional ecological landscape corridors. The present distribution situation of riverfront greenspaces had obvious characteristics of spatial differentiation between urban areas and SUAs. PG was the main type of greenspace in the urban area; CL and FL were the main types of greenspace in the suburbs and RA.

Type-5 rivers were located in the SUA where urban and rural areas intersect, with a width of more than 30 m. CL and PL were the dominant greenspace types. Most of the river segments mainly served agricultural production in the suburbs, while the PG type providing public leisure services was the main composition in the built-up areas.

Type-6 rivers were located in RA, with a width of more than 30 m. As an important water drainage channel and water source area, there are many tributary river networks in the surrounding area, so the ecological protection function of the water system is very important. The composition of the riverfront greenspace type was relatively singular. The demand for agricultural production and services in RA produced a large area proportion of CL types. Second, the FL or SRS type occupied a certain proportion, providing ecological protection for water sources and large-scale tourism and leisure space for metropolitan-area citizens.

### 3.3. The Spatial Quantified Factors and CI Effect of Riverfront Greenspace

The BRT model was used to estimate the interaction relationship between the morphological variables and the LST values of greenspace. The marginal effect (ME) of morphological factors on LST was analyzed, and the mechanism of each influencing factor was obtained.

#### 3.3.1. Morphological Influencing Factors of Greenspaces

**Figure 9:**
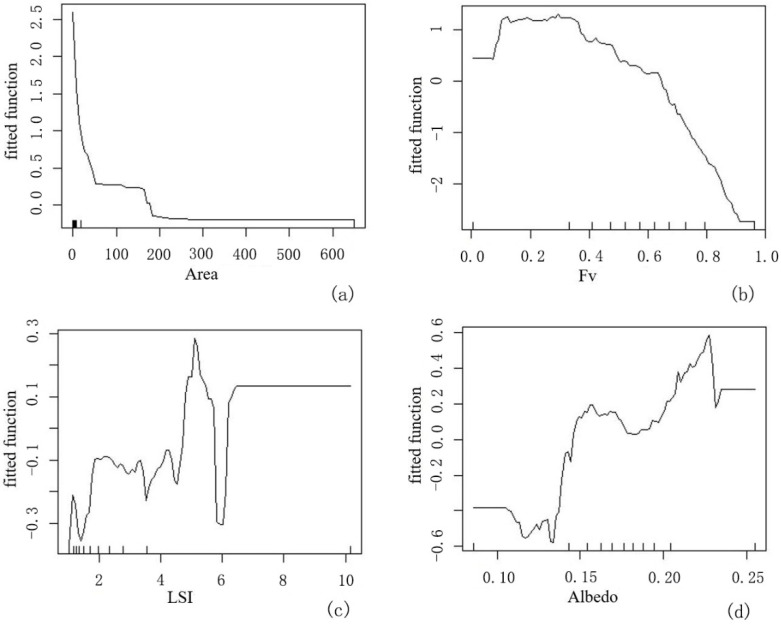
ME curve of morphological factors of greenspace on LST. (**a**) Fv of greenspace; (**b**) area of greenspace; (**c**) LSI; (**d**) surface albedo.

The morphological factors that affected the CI included the variables A, Fv, LSI, and albedo. The ME curve of A and LST showed a decreased inclination, and it was significantly negatively related to the ME of LST. Namely, with the increase in the area scale, the surface temperature of greenspace decreased. However, the influence of A on LST was obviously nonlinear, and there were certain threshold values (Figure 9a). The Fv variable had the largest impact on LST. The influence between them showed a significant negative correlation (Figure 9b). The overall ME curve of the LSI variable on LST showed an increased inclination, indicating that the shape index was positively correlated with temperature (Figure 9c). With the increase in the shape index, the LST of greenspaces increases gradually; the relationship between the surface albedo and LST of greenspaces is seldom complex. As in Shanghai, a study area with a dense water network, the influence of the soil aquifer has regional differences. The albedo values of green patches with better vegetation coverage were generally in the range of 0.16–0.19; under this condition, albedo and LST were negatively correlated, namely, with the increase in vegetation coverage, the surface albedo of green patches gradually increased. There was an exception: when the albedo was greater than 0.19, the type of greenspace corresponding to these albedo values included cultivated land and grassland types, resulting in a positive correlation between albedo and LST (Figure 9d).

#### 3.3.2. Spatial Structure Influencing Factors of Blue-Green Space

**Figure 10:**
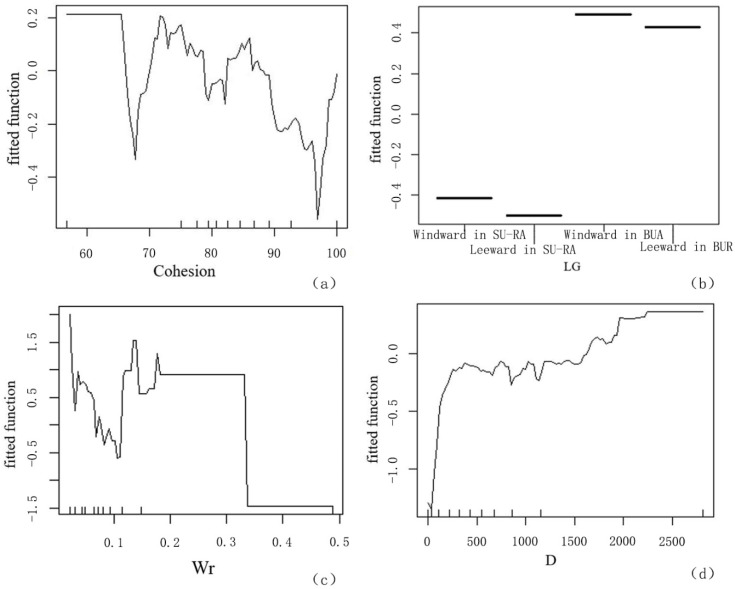
ME curve of structural factors of blue-green space on LST. (**a**) Distance from riverbank; (**b**) location; (**c**) Wr; (**d**) cohesion.

The spatial structural factors of blue-green space included the C, L, Wr, and D variables. The ME curve between the C variable and the LST mainly showed a decreased inclination, and the C values had a significant negative correlation with the LST of greenspace (Figure 10a). The LST of greenspace decreased with the increase in cohesion values; the L factor was a classification variable to determine the category values, so in the process of quantitative analysis, it represented little importance. However, the ME differences in LST corresponding to greenspaces in different orientations and positions were large, which indicated that they still had an important impact on the cooling intensity differentiation of riverfront greenspaces. In particular, the LST of green patches downwind was obviously lower than that of the upwind green patches (Figure 10b). The water surface area was composed of the water surface of the main backbone rivers and the surrounding small water bodies. In the BRT model calculation, all types of water bodies were summed up to the water surface area to explore the influence of water body distribution on CI. The ME curve of Wr generally showed a decreased inclination, and there was a significant negative correlation between Wr and LST (Figure 10c). The LST value distribution of waterfront greenspace was significantly affected by the D values. When the distances were less than 400 m, the D variable was significantly positively correlated with LST (Figure 10d). When the distances were more than 400 m, the difference in cooling distance caused by the difference in river width produced the fluctuation of the ME curve. When the distance was greater than 1500, the correlation between them was still positive. This numerical range is the effect of the D produced by the large main basin of the Huangpu River. With the increase in D, the LST increased significantly.

### 3.4. CI Characteristics of Different River Types

#### 3.4.1. Importance of Each Spatial Variable to the LST in Six River-Type Zonings

The BRT model was used to calculate the relative importance of each spatial factor of riverfront greenspace on LST values. The correlation analysis between the spatial quantified factors and LST of greenspace was conducted for the different river types, and the contribution ratio of each factor to LST was obtained. In the importance analysis of the impact of spatial quantitative factors on LST for six types of river classification, Wr was further divided into Wr-m and Wr-s to further distinguish the impact of rivers and surrounding regional waterbodies. The results are shown in Table 9.

Comparing the importance of the blue-green spatial pattern influencing factors on the greenspace CI in the riverfront area of six river types, the general character and difference of the CI dominant factors in different river types were concluded. The specific conclusions were as follows:

(1) In each type of riverfront area, the Fv and A factors provided a large contribution to the LST of greenspace. These two factors had a significant impact on the CI, and the composition and scale of greenspace were the dominant factors affecting CI intensity;
(2) The Wr and D factors had a significant impact on LST, which proved that waterbodies had an obvious effect on LST except for green coverage. The Wr-m was more related to the river width. In the same type of river, the width changes were relatively small, so the Wr-s factors more reflected the synergistic cooling impact of small waterbody in the surrounding area on greenspace. The denser the water network around the green patches was, the more obvious the influence of Wr-s on the CI of greenspaces;
(3) The influence of albedo was related to the spatial characteristics of the surrounding environment area. In the buffer areas of type-one and type-three rivers, the environmental interference of HDZ and the diversification of built-up space in NGZ made the albedo factors become significantly important to CI;
(4) In the type-four riverfront area, due to obvious urban-rural differentiation, different locations had prominent temperature gradient changes, which made the influence of the L factor prominent;
(5) In type-four and type-five riverfront areas, there were rural-urban fringe spatial pattern characteristics, CL and PG with continuous green belts coexisted, and cohesion values changed greatly. The C factor had an obvious effect on the CI of riverfront greenspace.

#### 3.4.2. Spatial composition Characteristics of the CI Pattern in Six River-Type Zonings

According to statistical analysis of the greenspace area proportions of different CI levels in the six types of river study areas, as shown in Table 10, it was found that, except for type-one and type-two rivers, which were seriously affected by the high-density built-up environment, the other four rivers have more than 10% strong cooling area with the level-six and level-five CIs; level-four, level-three and level-two CIs occupied the main area proportion at all levels of rivers. However, the insignificant CI accounts for a large proportion of the riverfront greenspace in the type-one to type-five river reaches. Only in the RA was the distribution proportion of type-six river reaches low. This characteristic indicated that the CI of riverfront greenspace in the built-up area was restricted to a certain extent. There were still a large number of low-efficiency garden greenspaces in riverfront areas from the perspective of climate adaptation.

**Figure 11:**
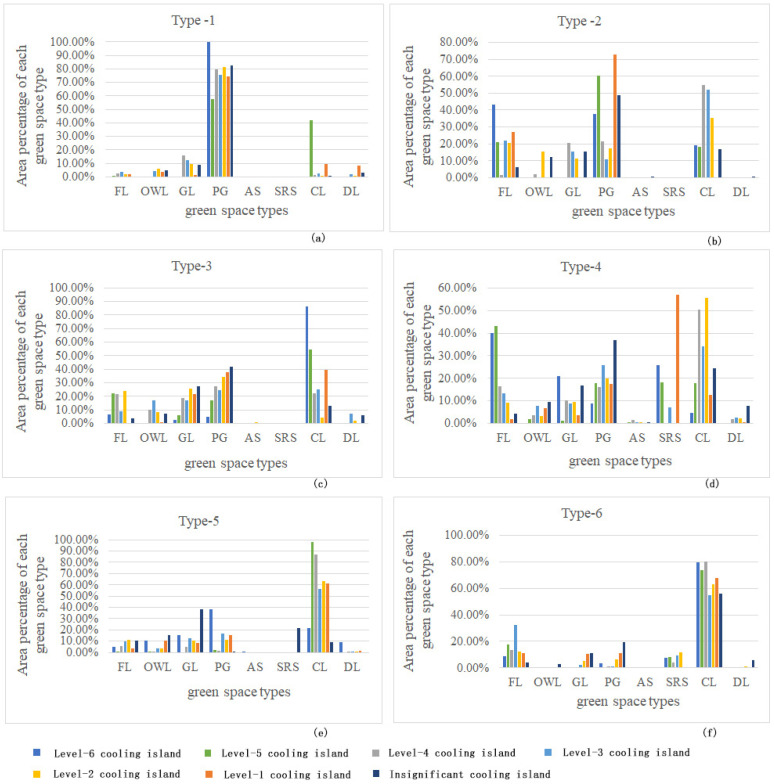
Area percentage of each greenspace type in the CI area with six different river types. (**a**) Type -1 rivers; (**b**) Type-2 rivers; (**c**) Type-3 rivers; (**d**) Type-4 rivers; (**e**) Type-5 rivers; (**f**) Type-6 rivers. Note: j in the composition of the one cooling island level which was represented by each color column, the sum of the area proportions of all greenspace types (FL, OWL, GL, PG, AS, SRS, CL, and DL) is 100%. Observing the same color column, it can be seen which greenspace type constitutes the main body of this cooling island intensity grade; k the various color columns of each type of greenspace indicate that the greenspace type has a different proportion of cooling island area. The closer the color column is to 100%, the more it reflects that the main cooling range of this greenspace type is located at the intensity level of the cooling island represented by the corresponding color.

By analyzing the area percentage of each greenspace type in the CI area at different levels (Figure 11), some issues of greenspace composition characteristics and spatial structure can be found, as shown below:

Type-1 rivers (Figure 11a): PG was the main greenspace type. All level-six Cis were composed of riverfront PG types, and in the level-five CI area, 57.75% of the area percentage was the PG type. The other area percentage of 41.92% was produced by CL type. Actually, the PG type accounted for the majority of Cis at all levels. Therefore, in high-density BUAs, the CI of greenspaces for public leisure services still had differences in scale and distribution pattern, and its holistic cooling intensity can be further improved by optimizing these two aspects.

Type-2 rivers (Figure 11b): PG, CL, FL, and GL were the dominant greenspace types. The corresponding greenspace types with strong cooling intensity, namely, level-six and level-five CI, were provided by FL, PG, and CL; however, approximately 72.91% of the greenspace area with relatively lower cooling intensity, namely, level-one CI, was the PG type. A certain proportion of FL types were also at this cooling level; an insignificant CI appeared in multiple greenspace types, especially PG types, which occupied 48.92% of the CI area. A large proportion of each greenspace type belonged to levels 2–4 with medium cooling intensity. With the further development of the NGZ, the space of CL and GL types accompanied by land-use transformation could become a key element for optimizing the cooling intensity of the riverfront area.

Type-3 rivers (Figure 11c): PG, CL, FL, and GL were still the dominant greenspace types. The areas with large proportions of level-six and level-five CI were the CL, FL, and PG types. Among them, the area proportion values of level-six CI were 85.99%, 6.59%, and 4.84%, respectively, and the area proportion values of level-five CI were 54.51%, 22.01%, and 17.13%, respectively. The main greenspace types of level-one CI were CL, PG, and GL. The main types of greenspace corresponding to the insignificant CI were the PG, GL, and DL types. The proportion of all types of greenspace in type-three with high CI intensity was relatively large. However, the strong cooling intensity was mainly provided by two types of CL and FL. In the process of developing new urban areas, it is very important to improve the green quality and cooling efficiency of the public recreational service system.

Type-4 rivers (Figure 11d): CL, PG, and FL were the dominant greenspace types, and the SRS type appeared in such river areas. The areas of level-six and level-five CI were mainly composed of FL, SRS, and PG types. Some CLs also had a certain proportion above level-five cooling intensity. The level-one CI was a plot of SRS and small-scale PG patches in the urban-rural fringe. The insignificant CI had a certain area proportion in many types of greenspace, especially in the DL, PG, and CL types. As the cooling intensity in the whole area was mostly above the level-two CI, this type of river had a better CI. The optimization of riverfront greenspace patterns in the urban-rural fringe is the crux to improving low cooling efficiency.

Type-5 rivers (Figure 11e): CL, PG, and GL were the dominant greenspace types. Level-six and level-five CI were essentially the distribution areas of CL type. The CL area covered by the level-six CI accounted for 76.89%, and in the level-five CI area, 97.72% of the area percentage was the CL type. The main greenspace types of level-one CI were small, isolated CL, PG, and ecological protection patches far from the river. All types had a certain proportion of insignificant Cis. Except for the suburban segments where the CL was located, green patches in other segments of the type-five rivers did not have good cooling efficiency, especially the FL and PG types. The improvement of the overall pattern of greenspace and green coverage quality in this type of riverfront area is urgently needed.

Type-6 rivers (Figure 11f): CL and FL were the dominant greenspace types. A large area of concentrated CLs and river surfaces can enhance the SCE in specific regional environments. The level-six and level-five Cis were also composed of FL and SRS with a certain area scale. Level-one and insignificant CI in this type of study area included most patches of GL, PG, CL, and DL. Type-six rivers were planned as ecological corridors that provide ecological and production services in RA. The intensity level of CI in riverfront greenspace had the coexistence of CI above level three and insignificant CI in large areas. Controlling the growth of land use and increasing the scale of ecological forestland can improve the ecological continuity and integrity of the greenspace for better cooling intensity in the river corridors.

## 4. Discussion

### 4.1. Maximizing the CI Efficiency Generated by Morphological Index Threshold

At the level of greenspace structure, within the scale of 50 ha, the larger the area is, the stronger the CI effect (Figure 9a). Therefore, in the limited urban construction space, the area scales of greenspaces reaching 50 ha should be considered in determining the optimal CI of large greenspaces. In addition, when the vegetation coverage of greenspaces was greater than 0.3, it developed a CI, and when it was greater than 0.62, it had developed a significantly strong CI (Figure 9b). Corresponding to the green-coverage index in urban planning, the green-coverage ratio of urban construction space should reach at least 30%. Parks and recreational public greenspaces with good thermal comfort should be controlled at no less than 62% green coverage in planning.

At the level of the blue-green network structure, the CI of other surrounding waterbodies was important. The larger the Wr of the surrounding environment, the stronger the SCE with the green patches. The Wr value reached 11%, resulting in the highest ME (Figure 10c). The construction of the blue-green network between the waterfront greenspace and the surrounding water body layout was the key work in providing riparian thermal comfort. The threshold value of the D factor was 400 m (Figure 10d). Reasonable organization of greenspace within 400 m offshore would have the largest synergistic effect of cooling. Therefore, the belt greenspace, PG, FL, and other greenspace types should be connected with each other to form an ecological-network landscape pattern and promote the SCE of waterfront cities on a large scale.

### 4.2. Spatial Morphology Indices Regularity Presented the Corresponding LST Interval

In the study area, various types of greenspaces have significant data interval differences in the aspects of the morphology index, which can more accurately describe the morphological characteristics of each greenspace patch. The regularity of greenspace types corresponding to the specific index data were as follows:

(1) Green patches with an area of less than 10 ha were mainly PG and AS types, and most were OWL, GL, and DL types. The greenspaces with 10–50 ha areas were mainly a large area of CL, FL, and GL, with a small proportion of medium-sized PG. The greenspaces with an area of more than 50 ha were mainly SRS, CL, and FL in the RA, with a small number of large parks (PG type);
(2) The green coverage ratio of FL, CL, and PL at large scales almost exceeded 65%. Apart from Pujiang Park in the south section of the N-S Huangpu River, which has low green coverage due to the large-scale construction of flower parks, the green coverage ratio of SRS exceeded 65%. The green coverage of municipal or industrial shelterbelts with small patch areas did not reach 65%. Small and medium-sized PG and AS were greenspaces with obvious hardening characteristics, and the vegetation coverages were basically between 30% and 65%. DL type, strip-shaped greenbelts along the riverbank and road, and small community parks had a green coverage ratio of less than 30%;
(3) The albedo values of most PG and other types of small green patches were less than 0.16; the albedo values of SRS and FL were generally between 0.16 and 0.19; the albedo values of most CL and GL types were more than 0.19;
(4) The LSI values of most regular PG, SRS, CL, AS, GL, and DL were less than 1.5; the LSI values of public green belts with large width, PG, SRS, FL, and CL with irregular boundaries were 1.5–3.0; and for narrow width of riparian PG and CL affected by town and village construction, their LSI values were greater than 3.0;
(5) The green patches of large FL, CL, SRS, and PG types had high cohesion values within 92–100%; the green patches of DL, GL, and OWL types mainly had medium aggregation degrees, with values between 82% and 92%; the green patches of small and medium-sized PG and AS had low cohesion values of less than 82%.

Based on factor ranks for the feature descriptions of greenspace types in Table 3, each type of greenspace had its own rank interval values of morphological indices (Table 11), which produced the differences in the CI intensity of riverfront greenspace. Once the FL type had high Fv, medium albedo, high cohesion, and low LSI, if its area was large, the LST of this green patch was low. The SRS type with high and medium Fv, high A scale, high cohesion, medium albedo, and low LSI was basically a low LST value area. AS and DL had opposite grades of morphology indices to the above two types of greenspace, which were basically LST high-value areas. The morphological index of CL had large-scale, high Fv, high cohesion, and low LSI in the RA; however, the albedo of CL was greater than 1.9. As mentioned above, the interaction between the albedo and LST of CL was positively correlated. Therefore, the CL type corresponded to a low LST value, but it was higher than the LST value under the same Fv condition of FL. In general, GL and OWL had high and medium Fv and high albedo, but they were small-scale, low or medium-cohesion, so the two types mostly belonged to higher distribution areas of LST values. The composition of PG is complex, and its indices also reflect a variety of morphologies. Among them, once-high Fv and albedo are essentially low and medium in this PG type, the LST would also have relatively low value patches, but most small patches and strip-shaped PG along the road were high LST value distribution areas.

### 4.3. The CI Pattern Correlated to Greenspace Type Composition at Six River-Type Zonings

Combined with the LST distribution characteristics of each greenspace type under the influence of spatial morphology indices, the main characteristics of CI-intensity distribution of the riverfront greenspace in each river-type zoning were further analyzed. The difference of CI effect caused by river types in different locations is obvious. It is obvious that there was interaction and correlation among service zoning, greenspace types, and CI pattern characteristics. The CI distribution characteristics caused by the influence of the differentiation characteristics of greenspace types are listed in Table 12.

### 4.4. Optimization Strategy of Riverfront Greenspace Based on Zoning Differentiation

Based on the current spatial characteristics of riverfront greenspace used for different functional zonings, the key influencing factors and optimization strategies of the blue-green space system oriented to CE were proposed as below.

For rivers in HDZ, the CI intensity of riverfront green patches was low, and there were a large number of insignificant CI green patches. In this type of riverfront area, the SCE of blue and greenspace was obviously affected by greenspace scale factor and coverage factor, albedo, and ecological connectivity. The specific optimization strategies are as follows: (1) Greening quality enhancing: Fv increased significantly after reducing the proportion of hard coverage area. For PG with more hard ground coverage, the greenspace coverage should be increased to more than 62%. (2) Albedo improvement: by increasing arbor vegetation to optimize the vegetation configuration. (3) C strengthening: by improving the proportion of riparian greenspace layout, the appropriate width, and green coverage of green belts along rivers and by strengthening the accessibility between rivers and green patches.

For rivers in NGZ, there existed a polarization distribution with high concentrated high-level CI intensity and very poor low-efficiency CI. Specially, the public leisure greenspace subsystem had a small area, low coverage and low albedo. The key factors influencing the CI intensity of riverfront greenspace in the zoning were A, Fv, and Wrs. For the river reach with very bad CE, the C index of the riparian green belt became another important key factor. The cooling optimization strategies are given based on these key factors: (1) increase greening area: by transforming GL into FL; increasing FL area and coverage by CL replacement to other locations; increasing PG area in the industrial park; (2) albedo improvement: by increasing the public green belt in the riverfront area flowing through the industrial land, and setting up a reasonable protective isolation green belt; (3) improve the influence of waterbody factors: by enhancing the width and continuity of the riparian green belt for strengthening the cooling distance and cooling intensity of the cooling source; reasonably planning public green corridors as the river reaches flowing through the industrial park to alleviate the thermal environment of the whole area; (4) C strengthening: by improving the proportion of riparian greenspace layout, the appropriate width, and green coverage of green belts along rivers and by strengthening the accessibility between rivers and green patches.

For the rivers of cross-districts, the CI intensity of riverfront greenspace in the built-up area was relatively low, and the blue–green space should further adjust the structure of the holistic cooling source circulation to enhance the CI intensity. The key influencing factors were Fv, C, Wrs, and L. The Fv and C distributions had obvious differences between urban and rural areas, and the PG had more hard ground coverage; the Wrs value was high, but the cooling efficiency of the riparian space was low. In the river segment of the urban-rural fringe area, the C value urgently needs to be improved. The cooling optimization strategy is as follows: (1) greening quality enhancing: by increasing greenspace coverage of PG type to more than 62%; (2) C increase: by improving the holistic connectivity of the riverfront greenspace, especially the connection between the scattered green patches in the built-up area and the surrounding waterbodies; (3) improve the influence of waterbody factors: by strengthening the scale of riparian greenspace and the numbers of parks and interlacing the greenspace network to enhance the SCE of rivers and greenspace; (4) concerning the L of greenspace: by adjusting the structural connectivity of green corridors between urban areas and RA to maximize the overall SCE.

For the rivers in SUA, the overall CI intensity level of riverfront greenspace was not high. The CE of riparian green belts was not significant. The key influencing factors are A, Fv, C, and Wrs. The A of greenspace was small and the Fv was low; the difference of C was obvious, the value range was medium, and the C value of PG was low; the CE of Wrs was relatively weak in the riparian areas. The cooling optimization strategy of these riverfront areas is given as follows: (1) greening quality enhancing: by transforming land use of GL and DL and optimizing the distribution of the PG and FL systems to improve the Fv of FL, PG, and OWL types; strengthening the construction of ecological isolation forest belts at the fringe of suburban towns to reduce the concentration of UHI; (2) C increase: by increasing large-scale park greenspace and forest park and riparian green belt to enhance the scale agglomeration effect, while providing ecological protection; and (3) improve the influence of waterbody factors: by rational planning of the riparian greenbelt and regional organization of connectivity to strengthen synergy CI.

For the rivers in RA, most riverfront greenspace had strong CE. The key influencing factors were Fv and Wrs. The proportion of CL type was too large, and the composition of greenspace type was relatively singular; except for Lianqi river in the north section, the Wrs were high, and the complex diversity of riparian areas needs to be emphasized. The cooling optimization strategy is proposed as follows: (1) greening quality enhancing: by increasing the recreational function types and FL type in riparian space, by improving the grid structure of ecological FL in the agricultural functional area, and by producing newly planned greenspace with higher Fv; and (2) improve the influence of waterbody factors: by optimizing the blue-green network structure to build a multifunctional composition landscape corridor network through combining with the characteristics of large Wr in this area.

### 4.5. Limitations of the Present Study

In this study, the interaction regulation between greenspace type and CI pattern were explored; this was used to explain that the internal influence mechanism, which was created by the differentiation of greenspace morphology and structure factors, resulted in the high and low differentiation of CI intensity. However, there is an inherent correlation between the diffusion and circulation of meteorological factors based on aerodynamics. Therefore, this can explain the differentiation characteristics with specific climate dynamic effects caused by the change of greenspace factors. In the current technical methods of Landsat image analysis of thermal environment effects, the data of such influencing factors as photosynthesis ratio, air temperature, wind speed, and humidity are not available, making this study limited in terms of the scientific interpretation of the impact mechanism.

In addition, this study only selected the LST data at a time when the UHI was in the high temperature period in summer to explore the interaction relationship between the multidimensional spatial characteristics of the blue-green space and the CI intensity. Studying the interaction between the CI and the spatial factors is complicated. Based on the interaction between meteorological factors and spatial-pattern factors, the seasonal changes of the synergistic CI effect of waterbodies and green patches need to be further studied. Future work can compare and analyze the different seasonal changes of the CI effect, so as to obtain a more comprehensive regulation of the blue–green space synergistic CI effect.

## 5. Conclusions

For support in forming a riverfront greenspace system adaptive to microclimate comfort in Shanghai, through quantitative analysis of RS, GIS, machine-learning algorithm, and mathematical statistics, the spatial differentiation characteristics of CI intensity under the influence of riverfront greenspace types in Shanghai were studied. The main research conclusions were as follows:

(1) The essence influencing CE factors of riverfront greenspace were Fv, area, Wr, D, LSI, C, and Albedo, which affect the CI intensity of blue–green space;
(2) Different types of greenspaces have their own morphological index characteristics, resulting in distribution differences in the CI intensity in riverfront greenspaces;
(3) The different river types had clear heterogeneity characteristic of greenspace types related to the functional zoning it serves, further resulting in the regularity of the CI distribution characteristics;
(4) Interactive relationships existed between riverfront greenspace types, river types, and the CI distribution characteristics. CI zoning characteristics, in serving function areas of river types, were produced by the composition and spatial morphology of each type of riverfront greenspace. Dominant characteristics of the riverfront greenspace type compositions related to the zoning functions of river types.

Based on the differentiation characteristics of greenspace types serving different zoning functions, taking greenspace as the research object, instead of the previous study on the differentiation of greenspace cold island pattern in riverside areas, which took grid units as the research object, this paper provides a new thinking method for the optimization of spatial subsystems at the subarea level, and formulates the development strategy of blue–green space in different zonings to slow down the heat island effect. Furthermore, this study provides a scientific basis for the optimization of the CI pattern of riverfront greenspace to different river types. Because the spatial scale of this study is mesoscale, the indices systems of greenspace composition factors have not been able to specifically describe the composition characteristics of vegetation components and three-dimensional green quantity. In the future, more diversified methods and tools can be used in the study process to describe the internal composition indices of greenspace and the impact of multiple microclimate indicators in blue–green space. Further refining the study on the pattern of spatial composition factors and microclimate factors can more specifically reflect the differences of local CI effects and expand the internal interactive influence mechanism and collaborative research methods that form the climate dynamics of blue–green space.

## Figures and Tables

**Table 1:** Classification system of greenspace in the urban riverfront area.

Greenspace Class	Greenspace Type	Definition and Description
Ecological conservation land	Forestland (FL)	It refers to forestland with tree canopy density greater than or equal to 0.2.
	Open woodland (OWL)	It refers to forestland with tree canopy density greater than or equal to 0.1 and less than 0.2.
	Grassland (GL)	It refers to the land dominated by herbaceous plants.
	Derelict land (DL)	It refers to the abandoned land with little or no vegetation cover.
Recreational greenspace	Park and greenbelt (PG)	Belt-shaped greenspaces and park systems with different grades for leisure and recreation as the main function.
	Afforested square (AS)	It refers to the land for independent squares with a vegetation-coverage ratio greater than or equal to 35% in the study area.
	Scenery recreation space (SRS)	Large greenspace with recreation and service facilities beyond the built-up area.
Agriculture use area	Cultivated land (CL)	It refers to the land for productive plantation, including paddy fields, vegetable fields, and other land for crop production.

**Table 2:** Multidimensional spatial variables to describe riverfront greenspaces morphology based on the CI.

Impact Variables	Selected Index	Definition and Description
Morphology of greenspaces	Area	Surface area occupied by the greenspace in units (m^2^).
	Fraction of the vegetation coverage (Fv)	Reflects the vertical coverage of the tree crown; the value of Fv ranges from 0 to 1.
	Landscape shape index (LSI)	Indicates the complexity of shapes, determined by calculating the deviation between the shape of the greenspace patch and a square of the same area.
	Surface albedo (Albedo)	Ratio of the surface reflection flux to the incident solar radiation flux on the surface of the greenspace. Corresponding data obtained through the Landsat 8 data retrieved through the ENVI5.3 software.
Spatial structure between blue and greenspaces	Cohesion	Described the connectivity of the blue-green ecological network. obtained by calculating in fragstats4.3 software.
	Location of the greenspace (L)	Defined according to the dominant wind direction position of the greenspace relative to the adjacent river and the location of greenspace in the urban or RA.
	Water surface ratio of main backbone rivers (Wr-m)	Percentage of the main rivers’ surface area adjacent to a greenspace in the area of the corresponding riverfront blocks.
	Small water surface ratio (Wr-s)	Percentage of small waterbodies and branch rivers around the greenspace in the area of the corresponding riverfront blocks.
	Distance to the riverbank (D)	Distance between the geometric center of the greenspace and riverbank, representing the influence of the waterbody on the CE of the greenspace.

**Table 3:** Classification of conventional spatial quantitative factors of greenspace.

Influence Factors	Classification Basis	Grade Interval	Factors Rank
Fv	Classified according to Fv threshold value of ME effect of cooling and the requirements of the local greenspace planning index. The classification is determined by combining the two standards.	0–0.3	Low
		0.3–0.65	Medium
		0.65–1	High
A	Classified according to the requirements of the greenspace planning index and the ME threshold of greenspace area.	<10ha	Small-scale
		10–50ha	Medium-scale
		>50ha	Large-scale
C	Divided according to the interaction between the spatial data distribution of the cohesion and the spatial differentiation of LST data.	<82	Low
		82~92	Medium
		>92	High
Albedo	Divided according to the reflection differentiation of the green patch surface types.	<0.16	Low
		0.16–0.19	Medium
		>0.19	High
LSI	Divided according to the regularity of the morphological boundary generated by the surrounding interference.	<1.5	Low
		1.5–3.0	Medium
		>3.0	High
Wr	Classified according to Fv threshold value of ME effect of cooling.	<0.06	Low
		0.06–0.11	Medium
		>0.11	High

**Table 4:** The composition of each riverfront greenspace type in CI regions with different levels.

Greenspace Types	The Area Ratio Composition of Each Greenspace Type in Different CI Level Regions							
	Level 6	Level 5	Level 4	Level 3	Level 2	Level 1	Insignificant	Type Proportion
FL	5.41%	29.22%	24.08%	24.67%	10.95%	0.64%	5.03%	100%
OWL	4.66%	2.62%	11.65%	14.56%	11.58%	5.41%	49.52%	100%
GL	3.06%	0.80%	19.57%	19.66%	18.52%	2.71%	35.67%	100%
DL	0.00%	0.00%	5.86%	11.63%	12.90%	1.48%	68.12%	100%
PG	1.84%	7.33%	11.12%	15.93%	18.08%	3.65%	42.07%	100%
AS	0.00%	1.16%	46.75%	7.66%	14.91%	0.00%	29.53%	100%
SRS	9.99%	32.99%	13.87%	20.34%	7.85%	14.95%	0.00%	100%
CL	4.23%	22.70%	34.21%	13.78%	14.36%	1.36%	9.36%	100%

**Table 5:** Area proportions of various greenspace types at different CI levels in the riverfront area.

Greenspace Types	Area Proportions of Various Greenspace Types at Different Level of CI							
	Level 6	Level 5	Level 4	Level 3	Level 2	Level 1	Insignificant	Type Proportion
FL	18.58%	21.71%	13.31%	21.74%	11.12%	3.66%	4.29%	14.47%
OWL	2.96%	0.36%	1.19%	2.38%	2.18%	5.72%	7.83%	2.68%
GL	5.14%	0.29%	5.29%	8.47%	9.19%	7.57%	14.88%	7.07%
DL	0.00%	0.00%	0.37%	1.18%	1.51%	0.98%	6.71%	1.67%
PG	6.37%	5.49%	6.19%	14.15%	18.51%	21.01%	36.22%	14.59%
AS	0.00%	0.01%	0.30%	0.08%	0.17%	0.00%	0.29%	0.17%
SRS	12.86%	9.19%	2.88%	6.72%	2.99%	32.04%	0.00%	5.43%
CL	54.08%	62.87%	70.47%	45.27%	54.33%	29.03%	29.79%	53.93%
Total value	100%	100%	100%	100%	100%	100%	100%	100%

**Table 6:** Area and area percentage of various types of riverfront greenspaces in the holistic river buffer area.

Landscape Types	Area (ha)	Area Proportion (%)
FL	8477.1705	14.7139
OWL	1545.4375	2.6824
GL	4184.4553	7.2630
DL	1208.0413	2.0968
PG	7760.4633	13.4699
AS	87.6673	0.1522
SRS	2549.9494	4.4260
CL	31,797.7848	55.1917
Total	57,613.3349	100.0000

**Table 7:** The composition characteristics of riverfront greenspaces in different river-type zoning.

River Function Classification	Names of Rivers of the Classification	Main Service Functions	Dominant Characteristics
Type-1: medium and small rivers in HDZ	Taopu creek, Dongjiao creek, Xisi creek, Nansi creek, Xinjing creek, Beiheng creek, Puhui creek	Public leisure and recreation service	Low Wr; PG was the main type of greenspace, and there were still large proportion of GL in some riparian sections.
Type-2: medium and small rivers in NGZ	Beimao creek, Liulei creek	Ecological protection and public leisure service	Low Wr; multiple types of greenspaces; PG, CL, and various ecological conservation service types.
Type-3: major rivers in NGZ	Chunshen creek, Yu creek	Public leisure and recreation service	Medium Wr; a large area proportion of greenspace was PG type; various ecological conservation service types and CL types also occupied a large proportion.
Type-4: major rivers of cross-districts	Yunzao river, Dianpu river, Suzhou river, N-S Huangpu river	Multifunctional ecological landscape and agricultural production services	The heterogeneity between urban and RAs, with high Wr in the suburbs and RA, reverse in built-up area; CL, PG, and FL were the dominant types of greenspace, and CL type was distributed in the suburb and RA.
Type-5: major rivers in SUA	Panjing creek, Dazhang creek, Zhuanxin creek	Agricultural production, ecological protection and public leisure service	Medium Wr; CL and PG were the dominant types of greenspace, GL and DL type also occupied a relatively large proportion.
Type-6: major rivers in RA	Lianqi creek, Youdun river, Lanlu gang river, W-E Huangpu river	Agricultural production and ecological protection service	High Wr; the area proportion of CL type was very high; FL and SRS also occupied a high proportion relative to the total respective types of area.

**Table 8:** Area percentages of various types of riverfront greenspaces at the river reach level.

River Classification	Names of Rivers	Area Percentage of Various Greenspace Types							
		FL	OWL	GL	DL	PG	AS	SRS	CL
Type-1 rivers	Taopu creek	1.31%	5.44%	20.45%	2.21%	70.60%	0.00%	0.00%	0.01%
	Dongjiao creek	0.00%	0.00%	0.00%	6.00%	94.00%	0.00%	0.00%	0.03%
	Xisi creek	1.83%	0.00%	0.00%	0.00%	98.17%	0.00%	0.00%	0.00%
	Nansi creek	0.00%	0.00%	0.00%	0.90%	99.10%	0.00%	0.00%	0.00%
	Xinjing creek	3.14%	0.86%	14.17%	0.00%	81.01%	0.00%	0.00%	3.90%
	Puhui creek	0.73%	10.70%	19.55%	3.40%	63.94%	0.00%	0.00%	1.68%
	Beiheng creek	3.22%	4.26%	1.63%	0.10%	65.97%	0.00%	0.00%	24.83%
Type-2 rivers	Beimao creek	12.49%	5.41%	11.91%	0.00%	30.42%	0.00%	0.00%	39.77%
	Liulei creek	11.82%	9.35%	16.74%	0.30%	39.88%	0.05%	0.00%	21.87%
Type-3 rivers	Chunshen creek	8.54%	8.05%	20.14%	9.47%	44.11%	0.00%	0.00%	9.68%
	Yu creek	12.97%	7.59%	18.09%	1.54%	23.83%	0.17%	0.00%	35.81%
Type-4 rivers	Suzhou river	10.60%	4.77%	5.93%	2.06%	17.96%	0.06%	0.00%	58.63%
	Dianpu river	18.10%	6.17%	9.54%	3.24%	16.67%	0.26%	4.71%	41.30%
	Yunzao river	19.00%	6.89%	7.66%	6.25%	24.41%	0.14%	11.11%	24.53%
	N-S Huangpu river	13.13%	5.29%	14.31%	1.58%	31.69%	1.02%	12.29%	20.68%
Type-5 rivers	Zhuanxin creek	1.89%	4.60%	4.67%	0.56%	25.26%	0.00%	0.00%	63.03%
	Panjing creek	2.93%	5.01%	14.07%	25.90%	25.90%	0.00%	0.00%	49.99%
	Dazhang creek	9.77%	5.86%	10.48%	4.74%	14.30%	0.20%	0.00%	54.65%
Type-6 rivers	Lianqi creek	6.37%	1.99%	4.27%	2.26%	8.29%	0.28%	21.61%	54.93%
	Youdun river	1.67%	0.62%	5.92%	0.68%	7.38%	0.00%	9.78%	73.95%
	Lanlu gang river	19.17%	0.12%	0.60%	0.29%	0.40%	0.00%	4.10%	75.31%
	W-E Huangpu river	21.62%	0.22%	0.75%	0.35%	2.46%	0.00%	3.16%	71.45%

**Table 9:** Relative importance of the spatial quantified factors to LST of riverfront greenspace in six river-type zonings.

Spatial Indices for Riverfront District	Relative Importance of Predictor Variables (%)					
	Type 1	Type 2	Type 3	Type 4	Type 5	Type 6
A	12.26	8.91	17.25	12.31	15.73	18.97
Fv	29.65	50.10	42.18	22.12	40.35	38.59
LSI	3.76	3.47	3.42	4.82	3.72	3.52
Albedo	14.82	4.22	9.48	6.55	4.76	5.85
L	2.60	3.61	4.39	9.45	1.30	2.62
Cohesion	4.47	4.10	4.81	17.13	8.76	3.50
Wr-m	6.52	7.73	6.17	3.91	7.20	11.15
Wr-s	13.55	9.96	2.58	13.11	9.58	8.41
D	12.37	7.90	9.72	10.60	8.60	7.39

**Table 10:** The greenspace area proportions of different CI levels in the six river-type zonings.

River Classification	The Area Ratio Composition of Different CI Levels							
	Level 6	Level 5	Level 4	Level 3	Level 2	Level 1	Insignificant	Total Ratio
Type-1	0.08%	8.92%	14.77%	17.17%	21.62%	4.44%	32.99%	100%
Type-2	1.35%	8.09%	15.14%	20.45%	10.62%	1.40%	42.94%	100%
Type-3	15.03%	7.77%	12.58%	17.83%	17.73%	5.29%	23.77%	100%
Type-4	3.22%	12.28%	17.01%	18.16%	24.36%	4.85%	20.11%	100%
Type-5	1.52%	9.23%	14.37%	16.85%	20.45%	3.55%	34.03%	100%
Type-6	8.09%	26.72%	34.77%	14.43%	6.83%	0.68%	8.48%	100%

**Table 11:** Morphological rank of various greenspace types corresponding to LST distribution in the riverfront area.

Landscape Types	Fv Rank	Area Rank	Cohesion Rank	Albedo Rank	LSI Rank	Main LST Interval
FL	High, Medium	Low, Medium, High	Mostly High, Medium, Low	Medium	Low, Medium	Low values
OWL	High, Medium	Low, little Medium	High, Medium	High, Medium, Low	Low, Medium	Higher value
GL	High, Medium	Low, Medium	Medium, Low	Mostly High, Medium	Low	Higher value, Lower value
DL	Low	Low, little Medium	Medium, High	High	Low	High value
PG	Low, Medium, High	Low, Medium, High	Low	Low, Medium, little High	High, Medium, Low	Low value, High value
AS	Low, Medium	Low	Low	Low, Medium	Low	High value
SRS	High, Medium	High	High	Medium	Low	Low value
CL	High, Medium	High, Medium, Low	High	High	Low, Medium, High	Lower value

**Table 12:** CI spatial characteristics of riverfront greenspace at six river-type zonings.

River Classification	Dominant Types	CI Pattern Characteristics Corresponding to Greenspace Type Composition
Type-1 rivers in HDZ	PG type	The grade of CIs of greenspace were mostly level-two and level-three, and there were a large number of green patches with insignificant CI.
Type-2 rivers in NGZ	PG, CL, and GL types	Polarization distribution of CI intensity, with high concentrated high-level CI intensity and very poor low-efficiency CI areas. The better CIs of blue-green space were mainly in level-two, level-three, level-four and level-five; The CI intensity of each type of greenspace needs to be improved.
Type-3 rivers in NGZ	PG, CL, and GL/FL types	The blue-green synergistic CI effect was relatively strong, and the high-value CI intensity dominated by CL and FL types. The cooling intensity of PG type needs to be improved.
Type-4 rivers of cross-districts	CL, PG, and FL types	Most of the green patches in the built-up area were level-two and level-three CI. The riparian space had some hard ground, and some greenbelt segments had a narrow width, which failed to maximize the SCE of blue–green space.
Type-5 rivers in SUA	CL, PG, and GL types	The overall CI intensity was not high. The greenspaces on both sides of some river segments were affected by the industrial land environment. There were large patches with no significant CI.
Type-6 rivers in RA	CL, SRS, and FL types	The CI effect in most areas of this river type were strong. The blue-green CI pattern had been relatively stable due to high Wrs and relatively good cohesion of the riparian greenbelts. The CI effect of PG in the central rural town needs to be improved.

## Data Availability

Not applicable.

## Abbreviations

LST	land surface temperature
UHI	urban heat island
CI	cooling island
SCE	synergistic cooling effect
CE	cooling effect
FL	forestland
OWL	open woodland
GL	grassland
DL	derelict land
PG	park and greenbelt
AS	afforested square
SRS	scenery recreation space
CL	cultivated land
HDZ	high-density built-up zoning
NGZ	new urban growth zoning
SUA	suburban areas
RA	rural areas
BRT	Boosted regression trees
ME	marginal effect
E-W	east-west orientation
S-N	south-north orientation
C	Cohesion
L	location of the greenspace
LSI	Landscape shape index
D	distance from river bank
Wr	water surface ratio
Wr-m	water surface ratio of main backbone rivers
Wr-s	small water surface ratio
A	area
Fv	Fraction of the vegetation coverage
SVF	sky view factor
Albedo	surface albedo
